# TSPAN7 Functions as an Antitumor Agent Through the STK11/AMPK/mTOR Axis in Colorectal Cancer

**DOI:** 10.1155/cjgh/5209381

**Published:** 2025-09-18

**Authors:** Tao Weng, Fenfen Hong

**Affiliations:** ^1^ Gastroenterology Department, Ningbo Yinzhou No. 2 Hospital, Ningbo, Zhejiang, China

**Keywords:** AMPK/mTOR, cell proliferation, colorectal cancer (CRC), STK11, *TSPAN7*

## Abstract

**Background:** Tetraspanin 7 (*TSPAN7*), a quadruple transmembrane glycoprotein, is involved in the growth, development, and energy metabolism of cells, particularly in tumor cells. Despite its recognized significance, the role of *TSPAN7* in colorectal cancer (CRC) remains unexplored. The purpose of this study is to explore the antitumor activity of *TSPAN7* through the STK11/AMPK/mTOR pathway.

**Methods:** Analysis of differential genes in normal colon and CRC tissues was conducted using the Gene Expression Omnibus (GEO) database. Ten potential genes, including *TSPAN7*, were also identified through the database screening.

**Results:** Assessment of *TSPAN7* alterations in both CRC tissues as well as cell lines revealed reduced expression as compared to normal colon tissues. Based on the findings, modulation of *TSPAN7* expression was performed through overexpression and downregulation in vitro within CRC cells. The findings indicated that *TSPAN7* exerted a negative regulatory influence on the migration and proliferation of CRC cells. An examination of AMPK and mTOR phosphorylation levels revealed that *TSPAN7* affected the phosphorylation of these proteins via STK11.

**Conclusion:** In CRC, *TSPAN7* exerts antitumor effects through the STK11/AMPK/mTOR axis.

## 1. Introduction

The global accessibility to modern medical care and an increase in the global average standard of life have resulted in significant advancements in the diagnosis and management of cancer [1]. However, the presence of unfavorable risk factors such as obesity, sedentary lifestyle, smoking, aging population, and dietary habits has led to a limited decrease in the occurrence and death rates of cancers, including colorectal cancer (CRC) [2, 3]. Since CRC only presents symptoms in advanced stages, there is a current need to identify new treatment targets to address the disease effectively [4].

Tetraspanin 7 (*TSPAN7*), a highly conserved quadruple transmembrane glycoprotein, possesses structural domains in the plasma membrane that can bind to several chaperone proteins [5]. The signaling pathway mediated by *TSPAN7* has the potential to influence the regulation of cell development, activation, growth, and motility of cell [6]. Previous research has indicated *TSPAN7* expression in gastrointestinal cancer cells, demonstrating its impact on the proliferation and invasion of hepatocellular carcinoma [7, 8]. However, there is currently a lack of evidence regarding the role of *TSPAN7* in CRC cells. Therefore, the objective of the present investigation was to explore the impact of *TSPAN7* on CRC proliferation and invasion and to elucidate the possible mechanism of action of *TSPAN7*.

## 2. Materials and Methods

### 2.1. Oncology Database Analysis

Transcriptomic data from CRC and normal colon tissues were retrieved from the Gene Expression Omnibus (GEO) database. The gene expression profile (GSE62646) was analyzed using R, following established criteria. Differentially expressed genes (DEGs) were identified and intersected with weighted gene co‐expression network analysis (WGCNA) results. A LASSO regression model, combined with the SVM‐RFE algorithm, was applied to select potential biomarkers, which were validated in clinical samples. Functional annotation of DEGs was performed using the R package “clusterProfiler” for GO, KEGG, and GSEA analysis (*p* < 0.05). GSEA was ranked by LogFC, with the top 10 enriched pathways visualized. Finally, bioinformatics analysis predicted the function of the potential biomarker, using criteria of *p* < 0.05 and fold change > 2.

### 2.2. Cell Culturing

The human CRC cell lines (Caco2, SW180, HCT116, and HT29) and the normal human colon cell line (NCM460) were sourced from ATCC (Manassas, VA, USA). All these cell lines were cultivated in DMEM medium (Gibco, Waltham, USA) supplemented with FBS (10%, Gibco) and maintained at 37°C in a 5% CO_2_ incubator.

### 2.3. Immunoblotting for Proteins

Tissues and cell lines were processed and lysed in a lysis buffer (RIPA buffer) containing PMSF at 4°C, followed by centrifugation with high speed at 12,000 × *g* for 15 min at a temperature of 4°C for the removal of cell debris. The final supernatant was collected and their total protein was quantified by the BCA protein assay kit. For immunoblotting, equal quantity (20 μg) of isolated proteins were first fractionated by 10% SDS polyacrylamide gel and then blotted to 0.22 μm PVDF membrane. After blocking, the membrane was incubated with primary antibodies at 4°C for overnight followed by a 2 h treatment with the appropriate secondary antibodies at room temperature (RT). Antibody staining was visualized through the enhanced chemiluminescence method.

The primary antibody information is as follows: Anti‐*TSPAN7* (Abcam, USA, ab211870), Anti‐mTOR (Abcam, USA, ab134903), Anti‐pAMPK (Abcam, USA, ab92701), Anti‐tAMPK (Abcam, USA, ab32047), Anti‐STK11 (Abcam, USA, ab199970), and Anti‐GAPDH (Abcam, USA, ab8245).

### 2.4. Immunohistochemistry Study

CRC tissues were fixed in 4% paraformaldehyde for 3‐4 h and cut into precoated slides at a thickness of 2 mm. After dehydration, dewaxing, and hydration, primary antibody was added to the slides at 4°C overnight according to the instructions, and then secondary antibody was added and photographed under the microscope.

### 2.5. Cell Viability Analysis

The viability of cells was determined by CCK‐8 assay. Briefly, 5000 cells per well were plated in 96‐well flat bottom plates followed by an addition of CCK‐8 assay reagent (1:10 of medium) at three different time points, including 24, 48, and 72 h. The cells were incubated for the duration of 1 h in the dark at RT. The color development was measured against the wavelength of 450 nm in a spectrophotometer. Cell viability calculations were performed by following the instructions provided in the reference.

### 2.6. Cell Scratch Assay

Briefly, the cells were plated into 6‐well flat bottom plates, and once the cell confluency reached 90%, the cell monolayer was gently scraped by using a sterile tip of 200 μL pipette to form a uniform trace along the center of each well. After forming scratch, the wells were washed three times with PBS for the removal of floating cells, and the medium was refreshed by the fresh medium. The cells were then placed for 24 h in a CO_2_ incubator. Images were taken at both the initial stage and after 24 h, using a microscope to assess the migration of cells into the injured area.

### 2.7. Cell Invasion Assay

The experiment was performed in a 24‐well, 8 μm transwell cell culture chamber equipped with Matrigel‐coated culture plates. Cells were digested using 0.25% EDTA trypsin. The upper chamber was loaded with 200 μL cells, while the lower half of the wells contained 600 μL of media with 10% serum. Following a standard 24 h incubation period, the cells were fixed in methanol for a period of 30 min and stained with Giemsa for an additional 30 min. The remaining cells in the upper chamber were carefully extracted and positioned under an inverted microscope to enable the counting of the remaining cells.

### 2.8. Cell Transfection

Cell transfection was conducted in a cell culture plate by seeding the desired quantity of cells into the wells and allowing them to attain a confluency of 50%. Once the desired confluency was reached, the cells underwent three washes with PBS. The siRNAs were designed using BLOCK‐iT RNAi Designer, targeting different regions of the TSPAN7 mRNA to ensure efficient knockdown. The sequences were verified for specificity by BLAST analysis to minimize off‐target effects. Chemically synthesized siRNAs were obtained from Ambion. A nontargeting siRNA was used as a negative control. Cells were transfected using Lipofectamine 3000, following the manufacturer’s protocol. The knockdown efficiency was confirmed by quantitative real‐time PCR (qRT‐PCR) and western blot 48 h post‐transfection. A solution of siRNA and Opti‐MEM (without FBS) was prepared with Lipofectamine 3000 with a final concentration of 100 nM. The prepared solution was then introduced to the cells and allowed to incubate for 6 h. Following incubation, the cell medium was refreshed by fresh media and the cells were further incubated for 48 h before further processing.

### 2.9. qRT‐PCR

Total RNA was extracted using the TRIzol reagent (Vazyme Biotechnology, Nanjing, China) following the manufacturer’s instructions. Reverse transcription was performed using a reverse transcription kit to synthesize cDNA. qRT‐PCR was conducted using SYBR Green Master Mix (Vazyme Biotechnology, Nanjing, China) on ABI 7900 System (Applied Biosystems, Foster City, CA, USA). Each reaction was conducted in triplicate, and gene expression changes were calculated using the 2−△△CT method. The primers used in this study were as follows:

 TSPAN7: Forward: 5′‐ CTG​GTT​GCT​GGT​CTG​GTC​TT ‐3′; Reverse: 5′‐ AGG​CTT​GTC​CTT​GTC​CTC​CT‐3′.

### 2.10. Statistical Analysis

All the results of this study were presented as mean ± SD. The results were statistically analyzed by GraphPad Prism 9 where Student’s *t*‐test was employed to compare two groups, while multiple groups were compared by using one‐way ANOVA. A *p* value < 0.05 was considered to be statistically significant.

## 3. Results

### 3.1. Bioinformatics

Transcriptomic analysis was carried out on 132 CRC patients and 54 control individuals using the GEO database. A total of 145 significantly differentially expressed mRNAs were identified, with 47 mRNAs showing upregulation and 98 mRNAs displaying downregulation. The differential expression of genes was visually represented through a volcano plot. These differentially expressed RNAs were used for the prognostic assessment of survival of CRC patients. Forty‐two mRNAs were associated with good or poor survival prognosis of CRC patients (Supporting Table 1). Forty‐two mRNAs were functionally enriched in GO, including molecular function, biological process, and cellular component categories, as well as the KEGG pathway (Figure 1). After analysis, it was observed that these mRNAs were significantly involved in DNA conformational changes, receptor regulatory activity, protein‐DNA complexes, and transcription factor activity. Among these differentially expressed mRNAs, 10 mRNAs were identified as significant: *CA1, CHP2, MMP1, CXCL8, HSD17B2, TSPAN7, MS4A12, SLC26A2, DHRS9,* and *HSD11B2*, and we screened the highest expression RNA, namely, *TSPAN7*, to investigate their role in CRC.

Figure 1Bioinformatics analysis screened 10 key marker genes. Transcriptomic analysis was carried out on 132 CRC patients and 54 control individuals using the GEO database. (a) Principal component analysis, (b) heat map, and (c) volcano plot visualizing all differentially expressed genes in GEO dataset. (d) Module–trait relationship. (e, f) LASSO (least absolute shrinkage and selection operator).

**Figure:**
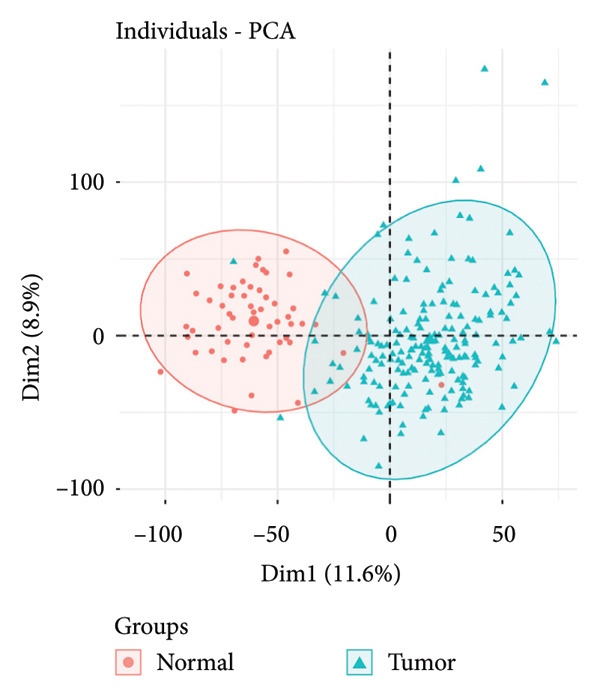
(a)

**Figure:**
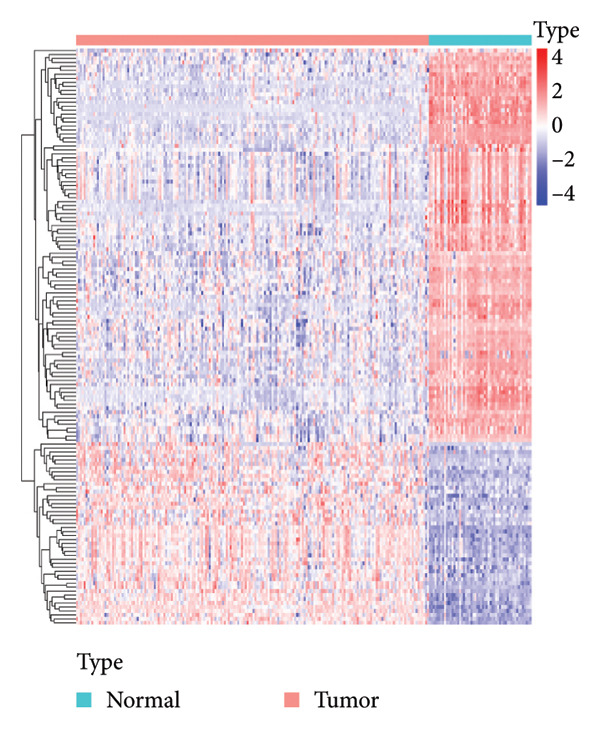
(b)

**Figure:**
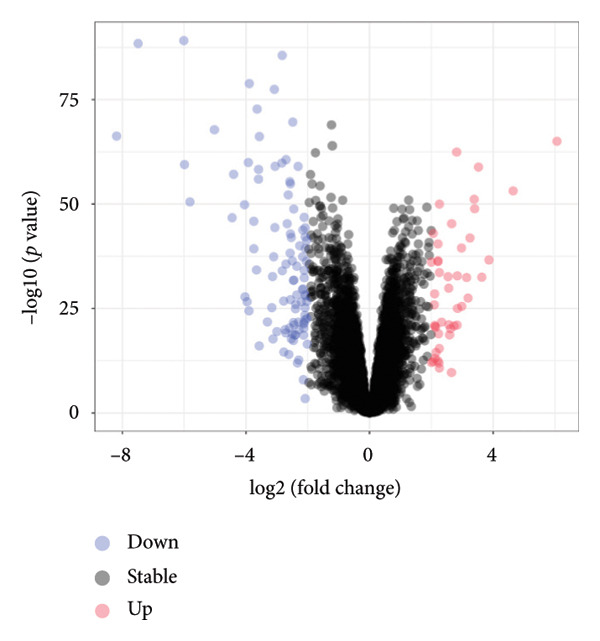
(c)

**Figure:**
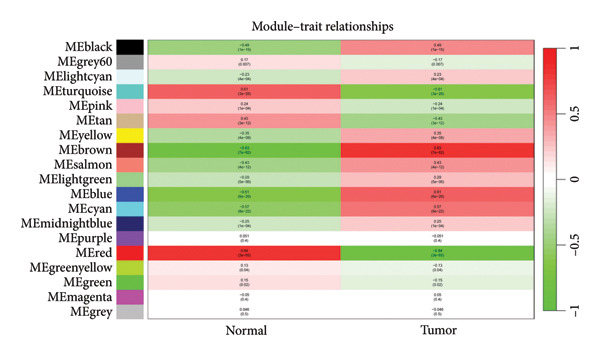
(d)

**Figure:**
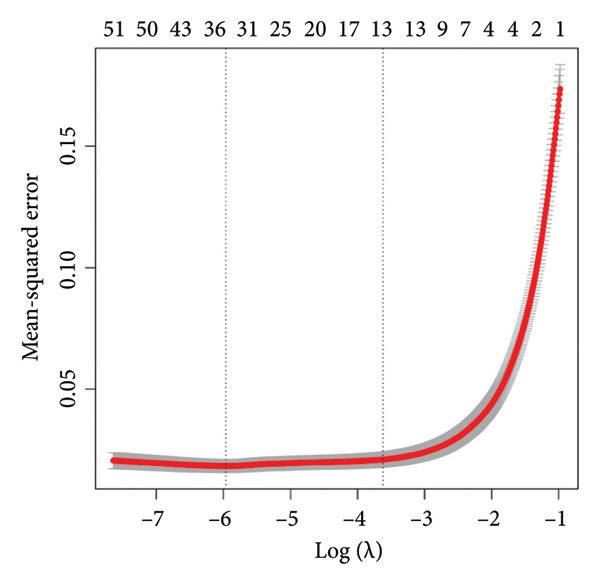
(e)

**Figure:**
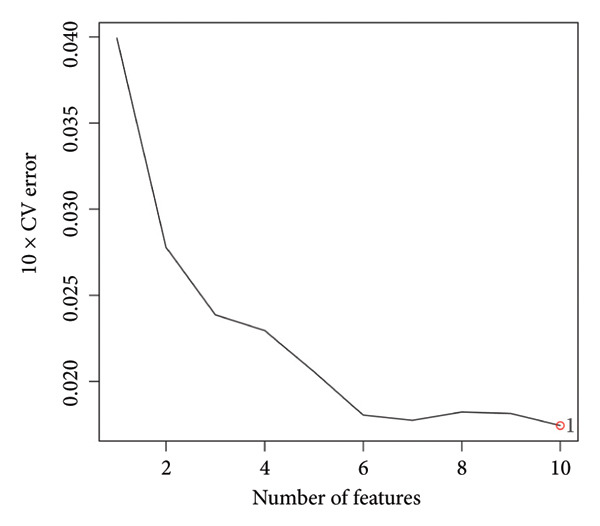
(f)

### 3.2. *TSPAN7* Expression Was Downregulated in CRC Cells

Gene expression analysis of normal colon and CRC tissues was analyzed by qRT‐PCR. The obtained results revealed that the mRNA expression of *TSPAN7* was downregulated significantly in CRC tissues compared with normal tissues (Figure 2(a)). Immunoblotting further confirmed a decrease in *TSPAN7* protein expression in CRC tissues (Figures 2(b) and 2(c)).

Figure 2TSPAN7 was downregulated in colorectal cancer patients and cell lines. (a) TSPAN7 mRNA expression is decreased in colon cancer tissue compared to normal tissue. (b) TSPAN7 protein expression is decreased in colon cancer tissue compared to normal tissue. (c) The statistical analysis of western blot for the expression of TSPAN7 in colon cancer tissue and normal tissue. (d) The expression of TSPAN7 mRNA was reduced in colorectal cancer cell lines (Caco2, SW480, HT29, and HCT116) compared to normal colorectal cells (NCM460). (e) The expression of TSPAN7 protein was reduced in colorectal cancer cell lines (Caco2, SW480, HT29, and HCT116) compared to normal colorectal cells (NCM460). (f) The statistical analysis of western blot for the expression of TSPAN7 in colorectal cancer cell lines (Caco2, SW480, HT29, and HCT116) and normal colorectal cells (NCM460). (g) IHC detection of TSPAN7 expression in normal tissues as well as colorectal cancer tissues. Black bar = 50 μm. (h) Survival curve analysis. ^∗^ *p* < 0.05, ^∗∗^ *p* < 0.01, and ^∗∗∗^ *p* < 0.0001.

**Figure:**
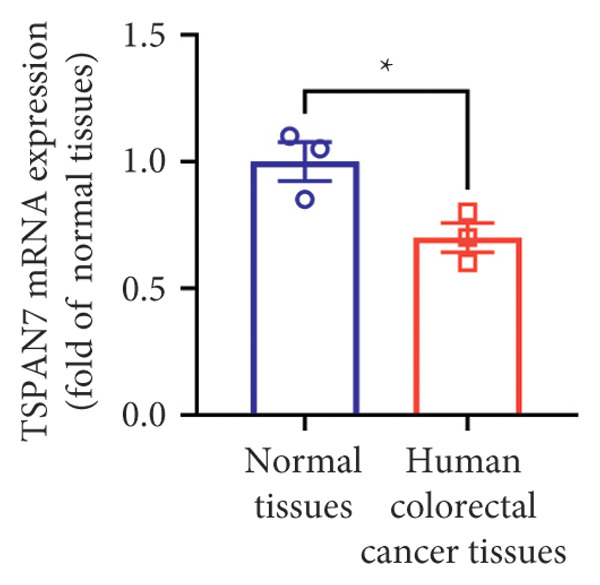
(a)

**Figure:**
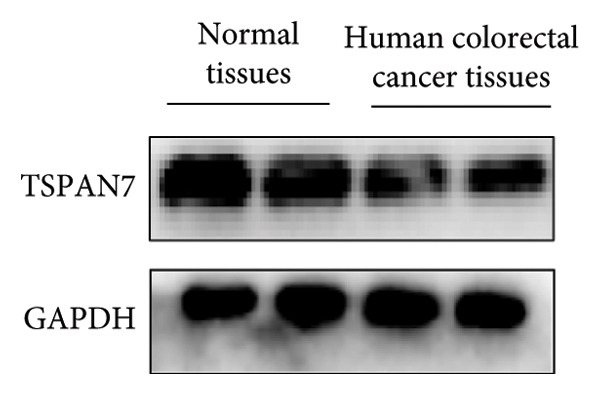
(b)

**Figure:**
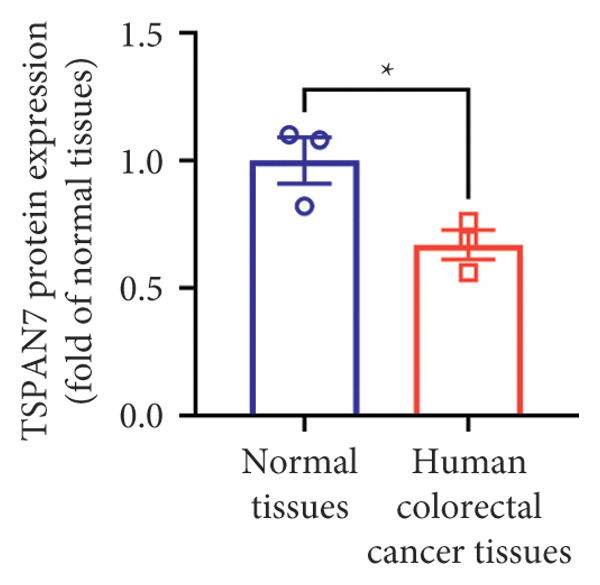
(c)

**Figure:**
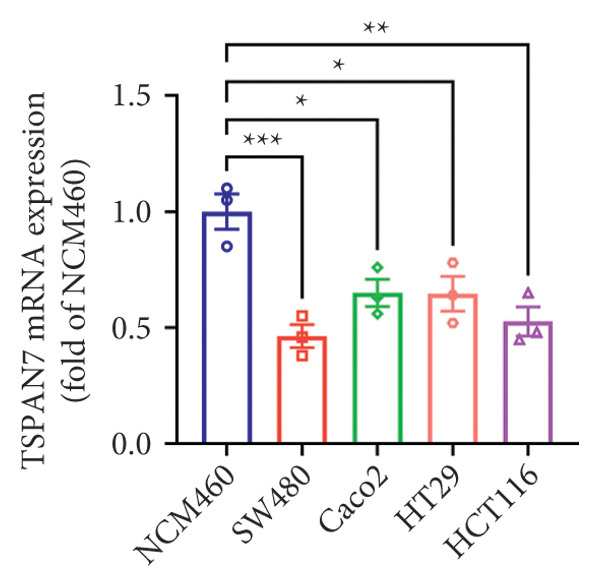
(d)

**Figure:**
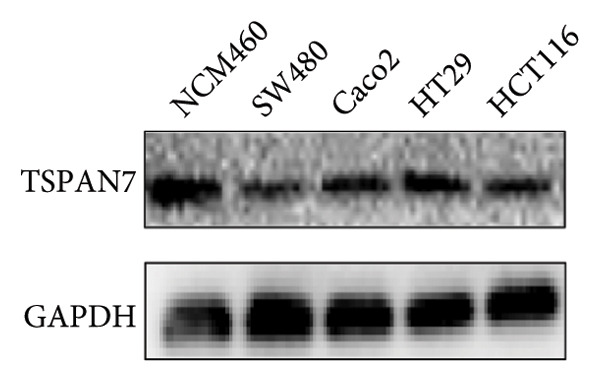
(e)

**Figure:**
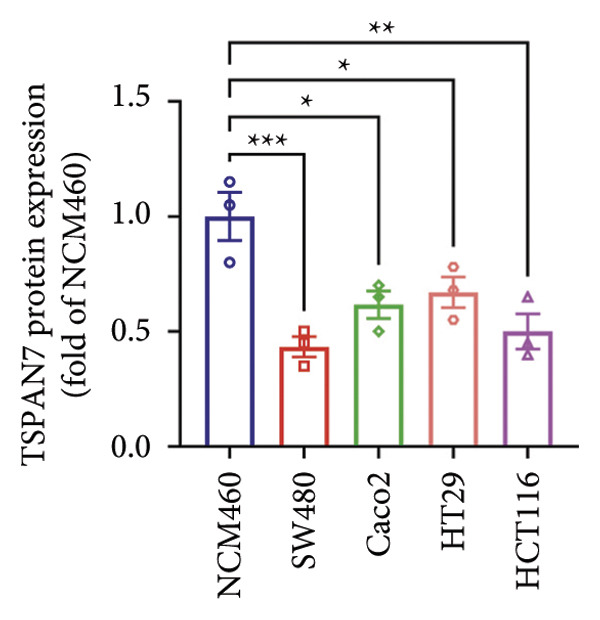
(f)

**Figure:**
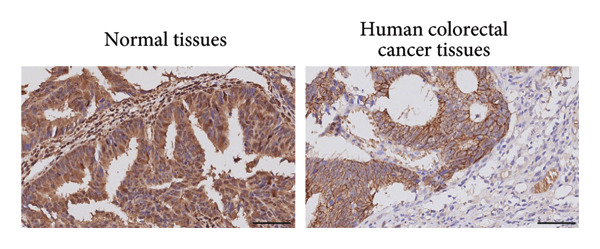
(g)

**Figure:**
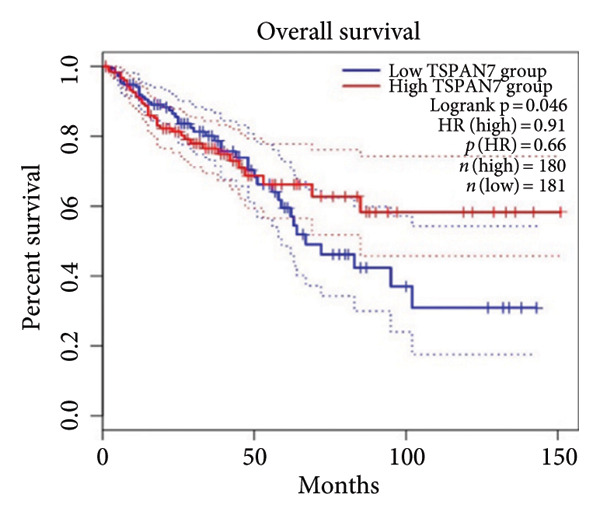
(h)

The in vitro analysis of *TSPAN7* expression in CRC (Caco2, SW480, HT29, and HCT116) cell lines produced results consistent with those observed in tissues. CRC cells displayed downregulation of both *TSPAN7* mRNA and protein compared to normal colorectal cells (NCM460). Both TSPAN7 mRNA and protein levels were consistently downregulated in the above CRC cell lines when compared with NCM460 (Figures 2(d), 2(e), and 2(f)). These results show that *TSPAN7* expression is downregulated in CRC cells. By IHC, we found that *TSPAN7* was also expressed at low levels in CRC cells and mainly distributed in the cytoplasm (Figure 2(g)). In addition, the lower the *TSPAN7* expression, the worse its prognosis and the lower its survival rate (Figure 2(h)).

### 3.3. Overexpression of TSPAN7 Inhibited CRC Cell Migration and Cell Proliferation

To further explore the potential influence of *TSPAN7* on the malignant behavior of CRC cells, the in vitro overexpression experiments were conducted using pcDNA‐*TSPAN7*. Results from RT‐PCR and immunoblotting demonstrated that pcDNA‐*TSPAN7* significantly upregulated the expression of *TSPAN7* in cells compared to the pcDNA group (Figures 3(a), 3(b), and 3(c)). Simultaneously, the proliferation of CRC cells was examined, revealing a significant reduction in proliferation capacity in cells overexpressing TSPAN7, as evidenced by the reduced cell viability over time (Figure 3(d)). Colony formation assays further demonstrated that TSPAN7 overexpression markedly decreased the number and size of colonies formed, highlighting its suppressive effect on long‐term proliferation (Figures 3(e) and 3(g), *p* < 0.01). Moreover, invasion assays confirmed that TSPAN7 overexpression effectively inhibited cell invasion, as indicated by the reduced number of invading cells observed under microscopy (Figures 3(f) and 3(h), *p* < 0.01). These findings collectively suggest that TSPAN7 plays a critical role in suppressing the proliferation and invasion of CRC cells.

Figure 3Overexpression of TSPAN7 inhibited cell proliferation and migration in SW480. (a) Verification of TSPAN7 overexpression efficacy at the mRNA level. (b) TSPAN7 protein expression was increased in SW480 when using pcDNA‐TSPAN7. (c) The statistical analysis of western blot for the expression of TSPAN7 in pcDNA‐vector group and pcDNA‐TSPAN7 group. (d) CCK‐8 assays and (e) colony formation assays confirmed that TSPAN7 overexpression reduced the proliferation capacity in SW480. (f) Transwell invasion assay and the statistical analysis confirmed that TSPAN7 overexpression inhibited cell invasion. (g, h) Statistical analysis. ^∗^ *p* < 0.05, ^∗∗^ *p* < 0.01, ^∗∗∗^ *p* < 0.0001, and ^∗∗∗∗^ *p* < 0.00001.

**Figure:**
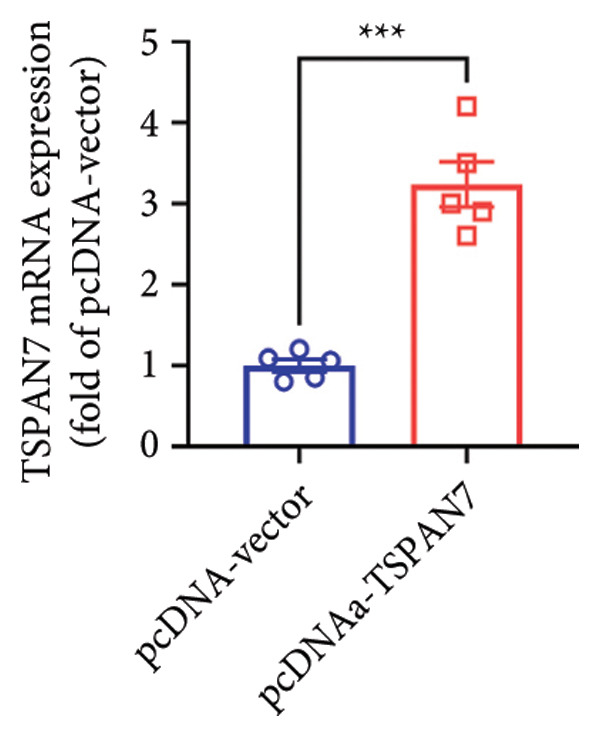
(a)

**Figure:**
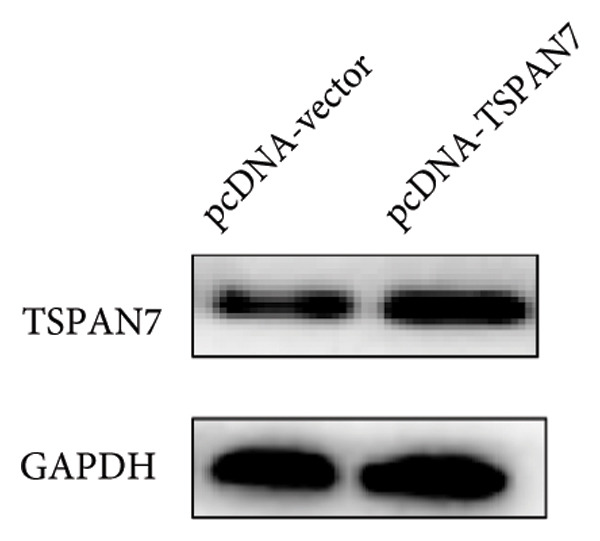
(b)

**Figure:**
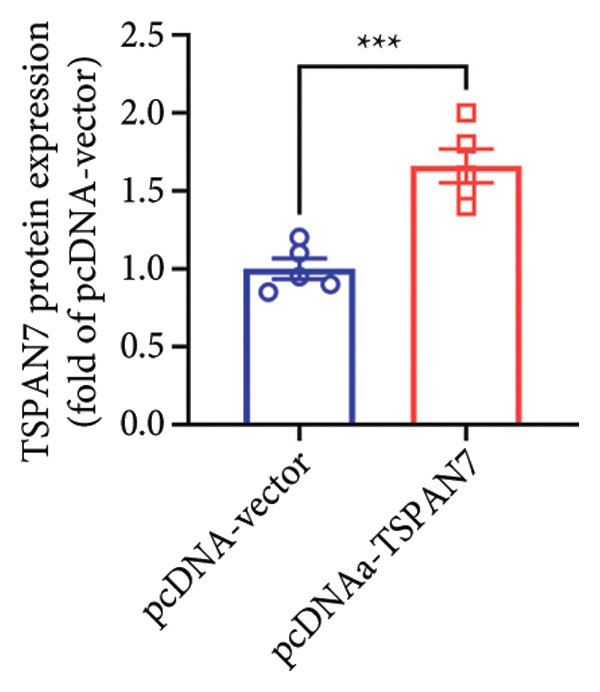
(c)

**Figure:**
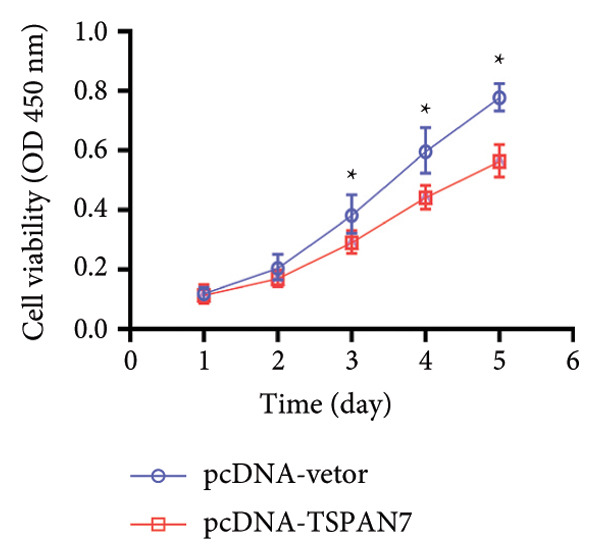
(d)

**Figure:**
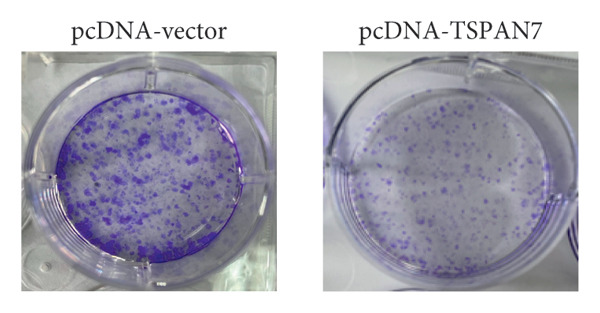
(e)

**Figure:**
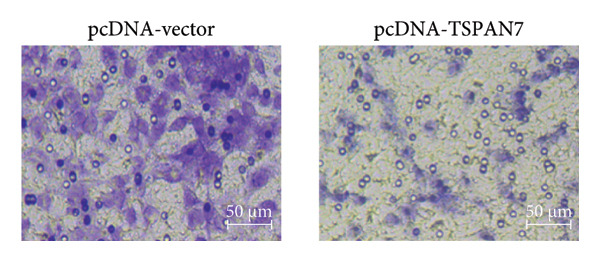
(f)

**Figure:**
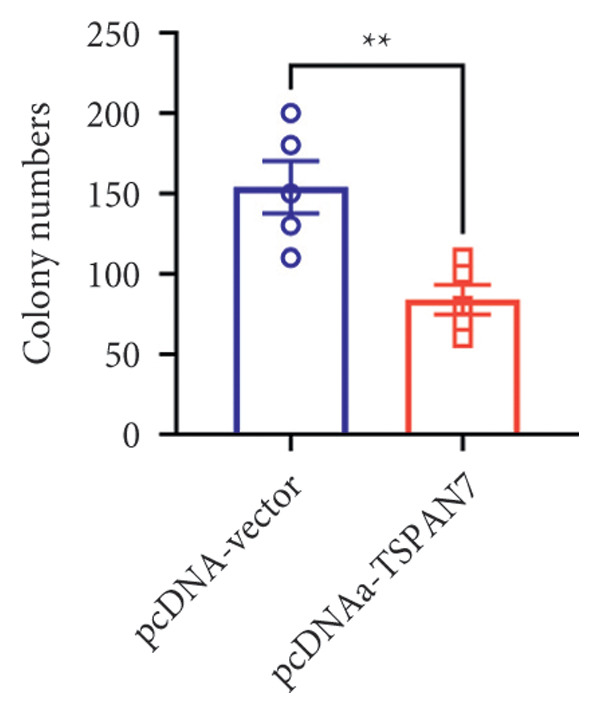
(g)

**Figure:**
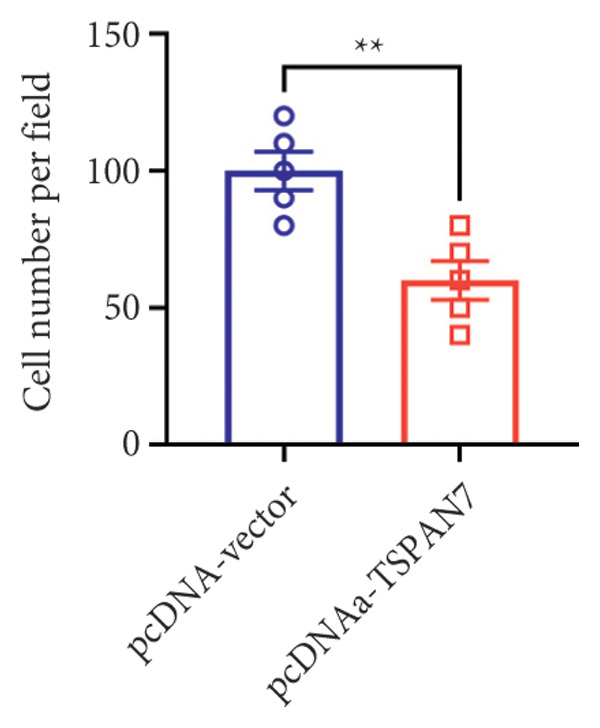
(h)

### 3.4. *TSPAN7* Inhibition Aggravated CRC Cell Migration and Proliferation

To find out the impact of reduced *TSPAN7* on the aggressive behavior of CRC cells, three siRNAs were designed for transfection into these cells. Immunoblotting and RT‐PCR were employed to assess the knockdown efficiency of these siRNAs. The results revealed that *TSPAN7* si‐2 showed the most effective knockdown efficiency, significantly reducing the expression of *TSPAN7* in CRC cells (Figures 4(a), 4(b), and 4(c)). An analysis of the proliferation of CRC cells after *TSPAN7* silencing revealed a significant increase in their capacity to proliferate (Figure 4(d)). The invasion assay further confirmed that overexpression of TSPAN7 reduced the malignancy of CRC cells (Figures 4(e), 4(f), 4(g), and 4(h)).

Figure 4Inhibition of TSPAN7 could aggravate the proliferation of colorectal cancer cells and migration in SW480. (a) Three siRNAs for transfection of SW480 reduced TSPAN7 mRNA level. (b) Representative western blotting images and (c) the statistical analysis for TSPAN7 protein in SW480 transfected into different siRNAs. (d) CCK‐8 assays confirmed that TSPAN7 inhibition using si2‐TSPAN7 promoted the proliferation capacity in SW480. (e) Colony formation assays and (f) statistical analysis confirmed that TSPAN7 inhibition using si2‐TSPAN7 promoted the migration capacity in SW480. (g) Transwell invasion assay and (h) the statistical analysis confirmed that TSPAN7 inhibition using si2‐TSPAN7 promoted the invasion capacity in SW480. ^∗^ *p* < 0.05, ^∗∗^ *p* < 0.01, and ^∗∗∗^ *p* < 0.0001.

**Figure:**
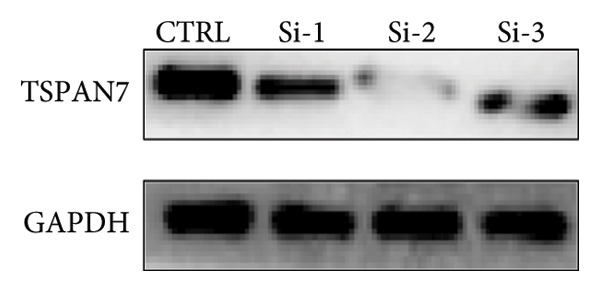
(a)

**Figure:**
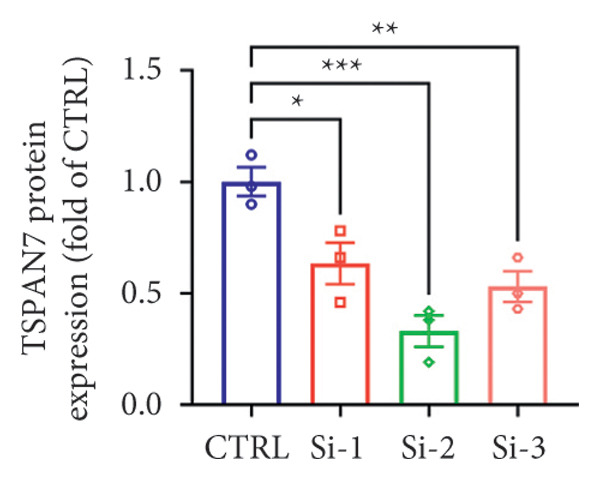
(b)

**Figure:**
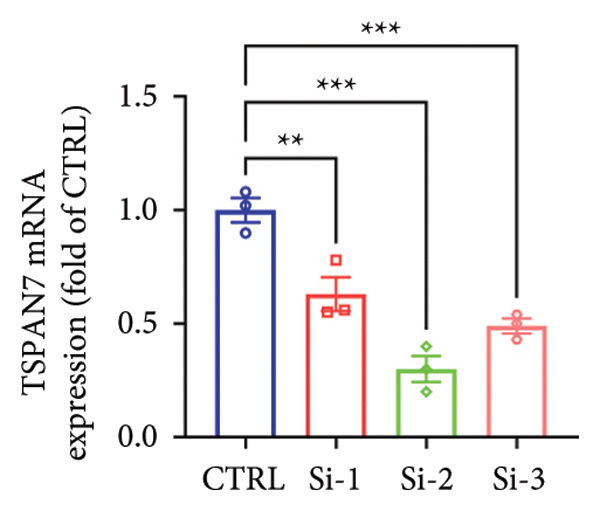
(c)

**Figure:**
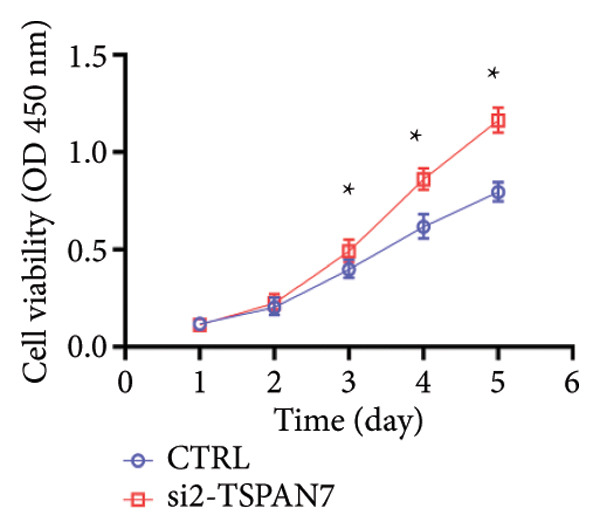
(d)

**Figure:**
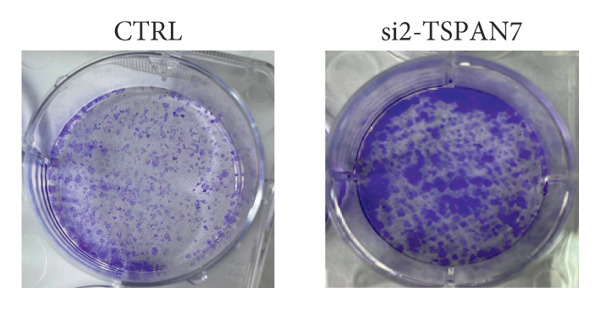
(e)

**Figure:**
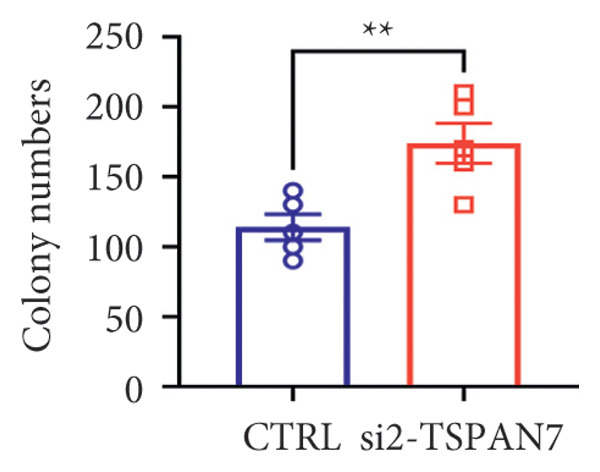
(f)

**Figure:**
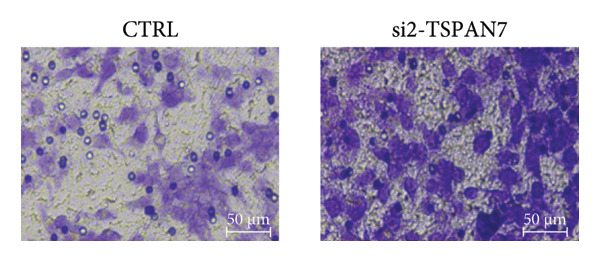
(g)

**Figure:**
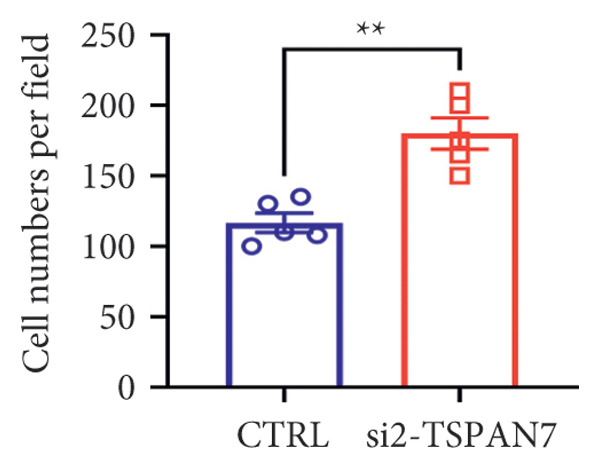
(h)

### 3.5. *TSPAN7* Inhibited CRC Cell Proliferation Through STK11/AMPK/mTOR Axis

In this study, we examined the effect of TSPAN7 overexpression on AMPK/mTOR signaling in CRC cells. We observed increased levels of phosphorylated AMPK (p‐AMPK) and upregulation of STK11 protein in TSPAN7‐overexpressing cells, suggesting that TSPAN7 may activate AMPK signaling via STK11. Although we did not directly assess phosphorylated mTOR (p‐mTOR) levels, total mTOR expression was decreased, implying a potential suppression of mTOR pathway activity.

The proliferation of tumor cells is closely linked to their metabolic activity, with STK11 playing a key molecule in this mechanism. To explore whether *TSPAN7* can influence tumor cell proliferation through STK11 [9], we treated TSPAN7‐overexpressing CRC cells with radicicol, a known STK11 inhibitor. Radicicol treatment significantly reduced p‐AMPK expression, consistent with inhibition of STK11 activity. Interestingly, this treatment also reversed the suppressive effects of TSPAN7 on CRC cell proliferation and enhanced cell invasion and migration abilities (Figure 5). These results suggest that TSPAN7 may exert its inhibitory effect on CRC progression at least in part through the STK11/AMPK pathway, although the involvement of mTOR signaling requires further confirmation.

Figure 5TSPAN7 inhibits the proliferation of colorectal cancer cell lines through the STK11/AMPK/mTOR axis. (a) Representative western blotting images and (b–f) the statistical analysis indicating that STK11 inhibition reversed the effects of TSPAN7 overexpression on mTOR, p‐AMPK, and STK11 protein expression. (g) STK11 inhibition reversed the inhibitory effect of overexpression TSPAN7 on migration capacity in SW480. (h) Transwell invasion assay and (i) the statistical analysis confirmed that STK11 inhibition reversed the inhibitory effect of overexpression of TSPAN7 on invasion ability in SW480. ^∗^ *p* < 0.05, ^∗∗^ *p* < 0.01, ^∗∗∗^ *p* < 0.0001, and ^∗∗∗∗^ *p* < 0.00001.

**Figure:**
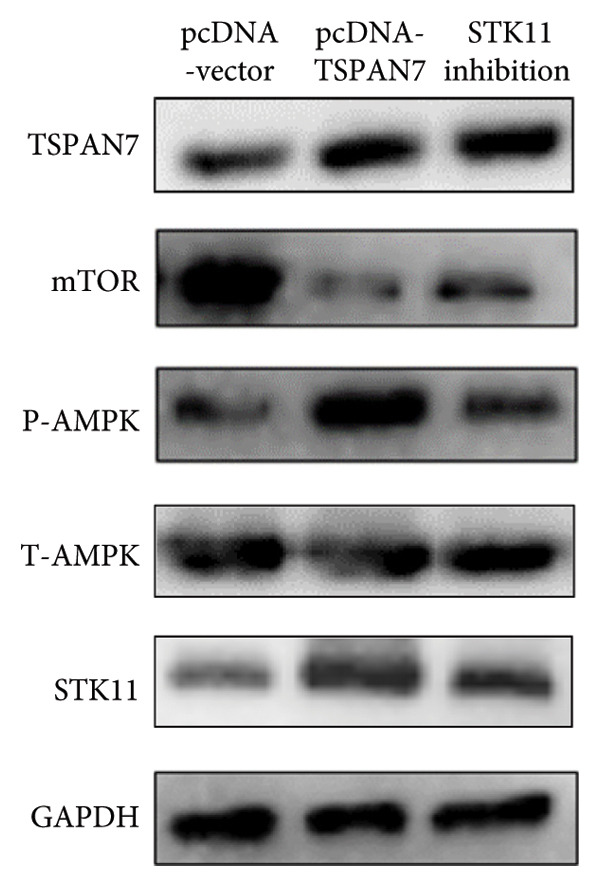
(a)

**Figure:**
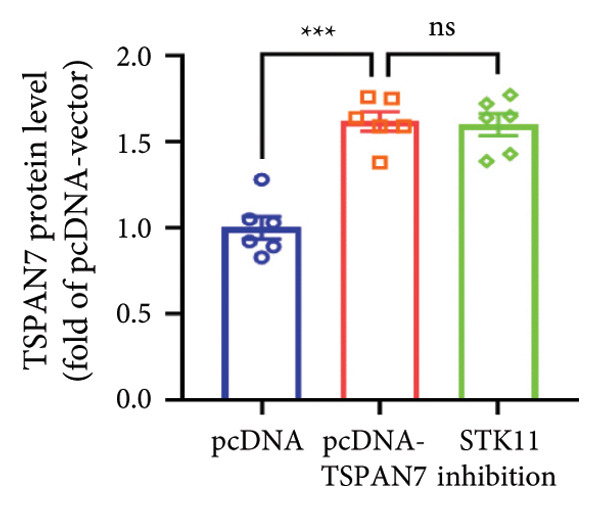
(b)

**Figure:**
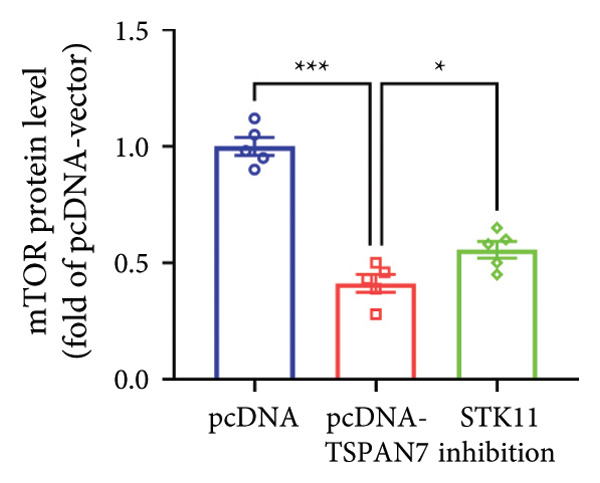
(c)

**Figure:**
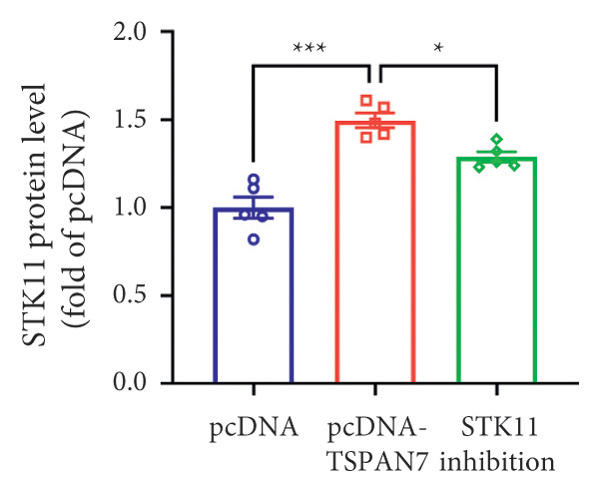
(d)

**Figure:**
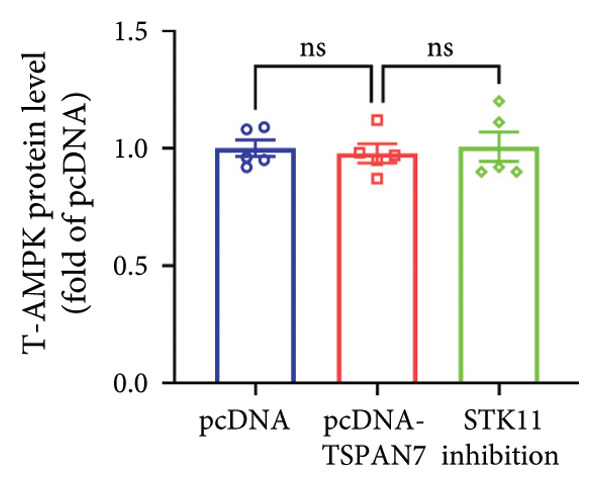
(e)

**Figure:**
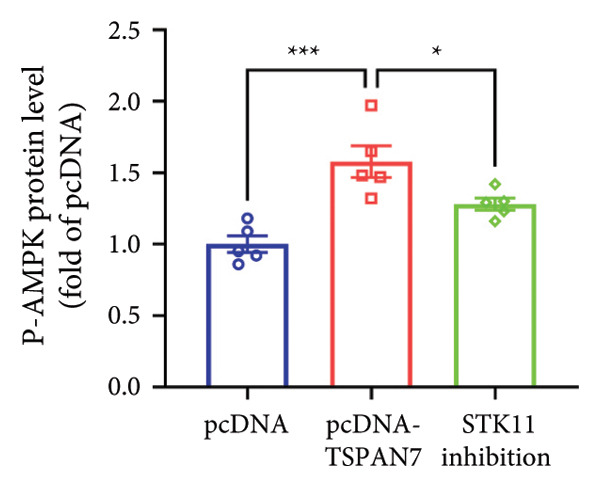
(f)

**Figure:**
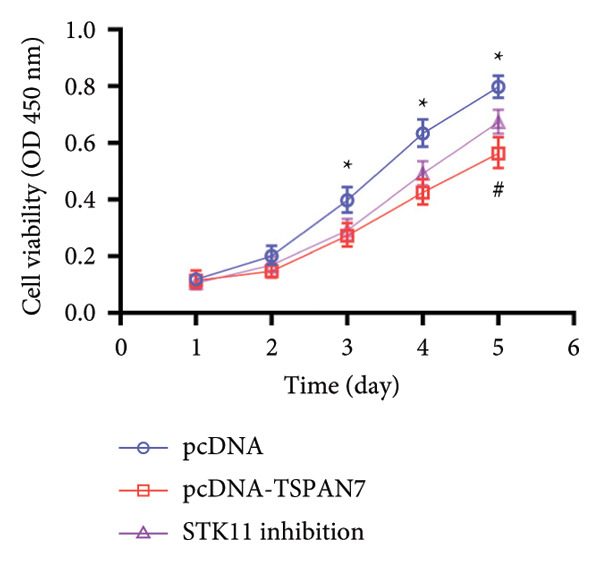
(g)

**Figure:**
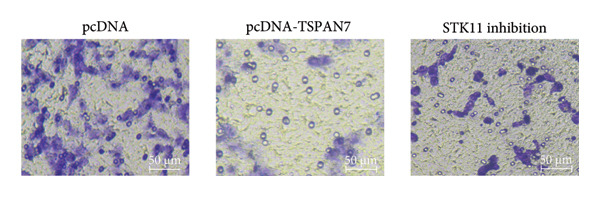
(h)

**Figure:**
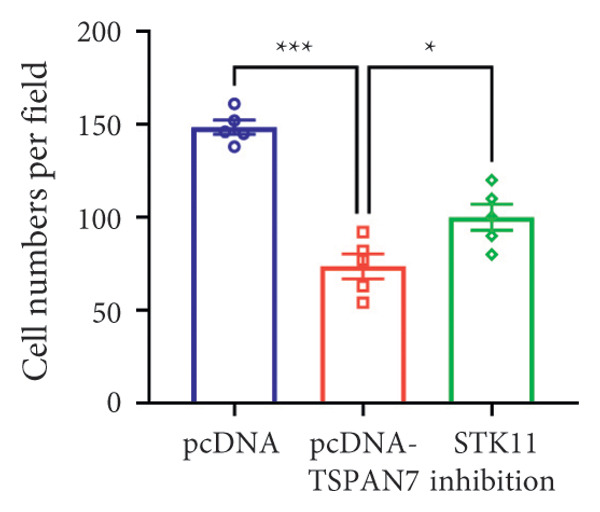
(i)

## 4. Discussion

The study revealed downregulation of *TSPAN7* in CRC cells, with correlation analysis showing a negative association between *TSPAN7*, proliferation, and migration of these cells. It was also found that *TSPAN7* affects CRC cell proliferation through the STK11/AMPK/mTOR axis. These findings introduce a novel target for the treatment of CRC.

In this study, low expression levels of *TSPAN7* in CRC were verified through bioinformatics and experimental analysis, marking the first confirmation of such findings. Several prior studies have reported the involvement of *TSPAN7* in various diseases [10–13]. The effect of *TSPAN7* on brain development involves the stimulation of axonal branching [14], whereas in pancreatic islet cells, *TSPAN7* controls Ca^2+^ channels which in turn affect insulin secretion [15]. Interestingly, *TSPAN7* is a double‐edged sword in tumor cells [16, 17]. In hepatocellular carcinoma, there is a positive correlation between TSPAN expression and patient prognosis [16]. Conversely, in lung cancer cells, its expression is inversely correlated with prognosis [17]. The current study postulated that *TSPAN7* is downregulated in CRC, and it is possible that similar mechanisms may exist in other gastrointestinal tumors, although further validation is required. The current investigation also demonstrated that upregulation of *TSPAN7* can suppress the growth and invasion of CRC cells. In the cell membrane, *TSPAN7* develops into a TSPAN network that is involved in cell motility, morphogenesis, and the interchange of materials and information within and outside the cell [5, 18]. Tumor cell activity primarily arises from changes in intracellular energy metabolism [19, 20]. By regulating the exchange of substances, *TSPAN7* influences the activity of tumor cells directly [5, 21].

AMPK is an essential kinase that regulates energy homeostasis, and its activation can lead to the downregulation of mTORC1 phosphorylation, affecting cell autophagy, apoptosis, and cell proliferation [22]. Several studies have reported that AMPK can inhibit the metabolic processes involved in tumor proliferation [23]. This study unveils a novel finding that *TSPAN7* influences the phosphorylation of AMPK under the regulation of STK11. This signaling protein serves as the upstream kinase, triggering the activation of AMPK protein in response to elevated AMP and ADP levels within cells. STK11 functions as part of an evolutionarily conserved energy‐sensing pathway linked to cellular metabolism and growth. It has also been reported as a critical player in tumor cells, inhibiting growth [24–27]. Therefore, the findings of this study showed that *TSPAN7* can inhibit the proliferation of CRC cell lines through the STK11/AMPK/mTOR axis.

Our study has several limitations. Firstly, the study provides strong in vitro evidence but lacks in vivo experiments using animal models, limiting the understanding of *TSPAN7*’s role in CRC within the tumor microenvironment. Secondly, the study focuses on the STK11/AMPK/mTOR axis, but other signaling pathways may also contribute to CRC progression, requiring further investigation. Thirdly, although radicicol was used to probe the STK11/AMPK/mTOR axis, it is not a specific inhibitor of STK11 and also targets HSP90, which can independently suppress mTOR signaling. Future studies should use more selective inhibitors or genetic tools to validate the upstream regulatory mechanism. Fourthly, this study was conducted in a single CRC cell line model, which may limit the generalizability of the findings. Future studies using multiple CRC cell lines and in vivo models are warranted to validate the role of TSPAN7 in CRC progression.

## 5. Conclusion

This study demonstrates that TSPAN7 is downregulated in CRC and inhibits tumor cell proliferation and invasion. These effects are at least partially mediated through the STK11/AMPK signaling axis. TSPAN7 may serve as a novel therapeutic target for CRC.

## Conflicts of Interest

The authors declare no conflicts of interest.

## Funding

The authors received no specific funding for this work.

## Supporting Information

Supporting Table 1 lists the 42 mRNAs associated with survival prognosis in colorectal cancer patients.

## Supporting information


**Supporting Information** Additional supporting information can be found online in the Supporting Information section.

## Data Availability

Publicly available data analyzed in this study were obtained from the Gene Expression Omnibus (GEO). All other data generated or analyzed during the current work are available from the corresponding author upon reasonable request for noncommercial academic use.
