# Supplementary material for: TSPAN7 Functions as an Antitumor Agent Through the STK11/AMPK/mTOR Axis in Colorectal Cancer
Source: Can J Gastroenterol Hepatol. 2025 Sep 18;2025:5209381. doi: 10.1155/cjgh/5209381 (PMC12445190; doi:10.1155/cjgh/5209381)
Supplement: Supplementary file 1 — Supporting Information Additional supporting information can be found online in the Supporting Information section. [file CJGH-2025-5209381-s001.docx]

***Supplementary Table 1 mRNAs associated with good or poor survival prognosis of CRC patients***

| Gene | GeneID | log2 Fold change | p-value | p-adj | |
| --- | --- | --- | --- | --- | --- |
| MMP1 | 4312 | 9.927 | 0.011068 | 0.39460 |  |
| CA1 | 759 | 6.041 | 0.001010 | 0.09935 |  |
| CHP2 | 63928 | 5.279 | 0.002560 | 0.17385 |  |
| CXCL8 | 3576 | 5.137 | 0.003697 | 0.21832 |  |
| DHRS9 | 10170 | 5.028 | 0.009524 | 0.36853 |  |
| SLC26A2 | 1836 | 4.662 | 0.016631 | 0.47954 |  |
| CA7 | 766 | 4.625 | 0.026762 | 0.61437 |  |
| UGT2B17 | 7367 | 4.536 | 0.033577 | 0.68533 |  |
| CA4 | 762 | 4.526 | 0.036557 | 0.71526 |  |
| CEACAM7 | 1087 | 4.517 | 0.023527 | 0.57344 |  |
| FABP1 | 2168 | 4.492 | 0.036423 | 0.71382 |  |
| MS4A12 | 54860 | 4.492 | 0.036423 | 0.71383 |  |
| CHD4 | 1108 | 4.479 | 0.024656 | 0.58486 |  |
| DHRS11 | 79154 | 4.441 | 0.026924 | 0.61521 |  |
| UGT2B15 | 7366 | 4.383 | 0.009809 | 0.37533 |  |
| MUC2 | 4583 | 4.370 | 0.034575 | 0.69508 |  |
| SLC4A4 | 8671 | 4.357 | 0.049662 | 0.79829 |  |
| SCNN1B | 6338 | 4.352 | 0.049775 | 0.79829 |  |
| CHI3L1 | 1116 | 4.330 | 0.036159 | 0.71207 |  |
| CA2 | 760 | 4.330 | 0.036159 | 0.71207 |  |
| ZG16 | 653808 | 4.279 | 0.042334 | 0.75692 |  |
| CLCA1 | 1179 | 4.264 | 0.041704 | 0.75062 |  |
| PLAC8 | 51316 | -4.230 | 0.044502 | 0.77134 |  |
| SLC | 44535 | -4.253 | 0.024133 | 0.57941 |  |
| CLCA4 | 22802 | -4.328 | 0.021823 | 0.55083 |  |
| TPH1 | 7166 | -4.360 | 0.018266 | 0.50335 |  |
| GCNT3 | 9245 | -4.360 | 0.018266 | 0.50335 |  |
| PMEPA1 | 56937 | -4.376 | 0.019615 | 0.52014 |  |
| KLK10 | 5655 | -4.382 | 0.032176 | 0.67372 |  |
| LRRC19 | 64922 | -4.392 | 0.018664 | 0.51018 |  |
| SLC26A3 | 1811 | -4.430 | 0.014903 | 0.45822 |  |
| NR3C2 | 4306 | -4.434 | 0.028413 | 0.63276 |  |
| AKR1B10 | 57016 | -4.439 | 0.014449 | 0.44752 |  |
| CLDN8 | 64922 | -4.446 | 0.026499 | 0.61009 |  |
| KRT20 | 54474 | -4.482 | 0.014085 | 0.44673 |  |
| HMGCS2 | 3158 | -4.503 | 0.013762 | 0.44179 |  |
| MUC4 | 4585 | -4.526 | 0.011423 | 0.40125 |  |
| HPGD | 64922 | -4.533 | 0.010951 | 0.39468 |  |
| GUCA2A | 2980 | -4.554 | 0.010648 | 0.39056 |  |
| FCGBP | 8857 | -4.742 | 0.006306 | 0.29549 |  |
| HSD11B2 | 3291 | -10.767 | 0.005463 | 0.27209 |  |
| TSPAN7 | 64922 | -12.5648 | 0.000254 | 0.03831 |  |
